# Use of electronic health data to identify patients with moderate-to-severe osteoarthritis of the hip and/or knee and inadequate response to pain medications

**DOI:** 10.1186/s12874-023-01964-y

**Published:** 2023-06-30

**Authors:** Yi Lu, Michael L. Ganz, Rebecca L. Robinson, Anthony J. Zagar, Sandra Okala, Craig T. Hartrick, Beth Johnston, Patricia Dorling, May Slim, Sheena Thakkar, Ariel Berger

**Affiliations:** 1 Evidera, London, UK; 2 Evidera, Waltham, MA USA; 3 Eli Lilly and Company, Indianapolis, IN USA; 4 Algosunesis, LLC, Bloomfield Hills, MI USA; 5 Pfizer Inc, New York, NY USA; 6 Duchesnay, QC Canada

**Keywords:** Osteoarthritis, Algorithms, Machine learning, Pain, Disease burden, Claims, Electronic medical records

## Abstract

**Background:**

No algorithms exist to identify important osteoarthritis (OA) patient subgroups (i.e., moderate-to-severe disease, inadequate response to pain treatments) in electronic healthcare data, possibly due to the complexity in defining these characteristics as well as the lack of relevant measures in these data sources. We developed and validated algorithms intended for use with claims and/or electronic medical records (EMR) to identify these patient subgroups.

**Methods:**

We obtained claims, EMR, and chart data from two integrated delivery networks. Chart data were used to identify the presence or absence of the three relevant OA-related characteristics (OA of the hip and/or knee, moderate-to-severe disease, inadequate/intolerable response to at least two pain-related medications); the resulting classification served as the benchmark for algorithm validation. We developed two sets of case-identification algorithms: one based on a literature review and clinical input (predefined algorithms), and another using machine learning (ML) methods (logistic regression, classification and regression tree, random forest). Patient classifications based on these algorithms were compared and validated against the chart data.

**Results:**

We sampled and analyzed 571 adult patients, of whom 519 had OA of hip and/or knee, 489 had moderate-to-severe OA, and 431 had inadequate response to at least two pain medications. Individual predefined algorithms had high positive predictive values (all PPVs ≥ 0.83) for identifying each of these OA characteristics, but low negative predictive values (all NPVs between 0.16–0.54) and sometimes low sensitivity; their sensitivity and specificity for identifying patients with all three characteristics was 0.95 and 0.26, respectively (NPV 0.65, PPV 0.78, accuracy 0.77). ML-derived algorithms performed better in identifying this patient subgroup (range: sensitivity 0.77–0.86, specificity 0.66–0.75, PPV 0.88–0.92, NPV 0.47–0.62, accuracy 0.75–0.83).

**Conclusions:**

Predefined algorithms adequately identified OA characteristics of interest, but more sophisticated ML-based methods better differentiated between levels of disease severity and identified patients with inadequate response to analgesics. The ML methods performed well, yielding high PPV, NPV, sensitivity, specificity, and accuracy using either claims or EMR data. Use of these algorithms may expand the ability of real-world data to address questions of interest in this underserved patient population.

**Supplementary Information:**

The online version contains supplementary material available at 10.1186/s12874-023-01964-y.

## Background

Osteoarthritis (OA) is a chronic, debilitating, degenerative disease that impacts over 30 million people in the United States (US) [1, 2]. It is a leading cause of chronic pain, and adversely impacts quality of life, activities of daily living, and health-related costs [3, 4]. These substantial impacts on health and life are more likely to be experienced disproportionately by those with moderate-to-severe disease and/or with relatively high levels of pain than other patients with OA [5, 6]. Relative to those with mild disease, patients with moderate-to-severe OA have more comorbidities (e.g., sleep disturbance, depression, anxiety); have poorer health status, health-related quality of life, and productivity; and experience higher levels of medication use and greater dissatisfaction with their medications [3]. Over 80% of patients with moderate-to-severe OA report experiencing daily pain versus 48.8% of those with mild OA [3]. Therefore, it is important to characterize and understand patient subgroups that have more severe disease and for whom currently used pain-related therapies are inadequate. 

One of the most efficient methods for conducting such studies would be to use large electronic healthcare databases that include information on the use of healthcare services and associated costs, as these sources tend to be relatively inexpensive to acquire and are fairly generalizable. Unfortunately, such databases typically lack the clinical information required to appropriately identify relevant OA subgroups (e.g., according to disease severity or response to pain-related medications). Conversely, medical records provide clinical detail, but lack complete data on use and cost of care; studies based on analyses of information extracted from medical records also tend to be relatively small in scope due to their expense and time required to collect necessary data. 

Previous studies have shown that predefined algorithms, based on expert knowledge and opinion, can adequately identify patients with hip/knee OA, with associated positive predictive values (PPVs) ranging from 0.61 to 1.00 [7–9]. However, we are unaware of algorithms that can identify important OA patient subgroups (e.g., moderate-to-severe OA, inadequate response to pain treatments), possibly due to the complexity in defining these characteristics as well as the lack of relevant measures (e.g., pain scores) in electronic healthcare data [10, 11]. We designed this study to address these challenges by developing algorithms intended for use with electronic healthcare claims and electronic medical records (EMR) databases that can identify patients with moderate-to-severe OA and inadequate response to pain medications. We developed several algorithms, including those based on existing knowledge of the OA disease process and others estimated using machine learning (ML) methods.

## Methods

### Data sources

We used healthcare claims and EMR data from two US integrated delivery networks (IDNs): the Henry Ford Health System (“HFHS”) and Reliant Medical Group (“Reliant”). Each system provided electronic versions of administrative healthcare data (including claims and eligibility data) and curated/structured EMR data, supplemented with information extracted from patients’ medical charts from the most recent 18 months available between January 1, 2015 and December 31, 2019 (“study period”) and prior to any knee/hip arthroplasty for which data were available at the time of study execution. Institutional Review Board approval was obtained from HFHS (IRB No: 13695) and Reliant (IRB No: 2609) before commencing the study.

HFHS is a comprehensive, integrated, non-profit health system that offers primary and acute care and specialty services to approximately 800,000 residents in the metropolitan Detroit area. Data available from HFHS include administrative claims (from patients covered by the Health Alliance Plan [HAP], which is HFHS’ insurance plan) and EMR data that are available regardless of insurance plan. HAP has approximately 650,000 enrollees, one-third of whom are aged ≥ 60 years. Reliant is a large, private, multi-specialty group practice in central Massachusetts with > 250 physicians in > 20 locations that collectively provide comprehensive care; they average more than 1 million patient visits annually. Reliant uses a comprehensive EMR system that captures data on ambulatory care, prescriptions, laboratory assessments, and radiology. Reliant has access to external medical and prescription claims data for 60% of its patients who are under capitated health insurance contracts. Available data include patients’ demographic characteristics, monthly enrollment history, medical and pharmacy claims, and laboratory results. 

### Patient selection

We included patients who were ≥ 18 years of age as of January 1, 2015 and who satisfied the following criteria during the study period: (1) had one encounter resulting in a diagnosis code of OA of the hip/knee (International Classification of Disease Version 9 Clinical Modification [ICD-9-CM]: 715.15, 715.25, 715.35, 715.95, 715.16, 715.26, 715.36, 715.96; International Classification of Disease Version 10 Clinical Modification [ICD-10-CM]: M16.x, M17.x) and (2) no evidence of cancer (except for carcinoma in situ or non-metastatic melanoma) at any time during the study period. 

We used the Clopper-Pearson (exact) method to estimate the required sample size assuming a PPV of 0.80 for the resulting algorithms. Results of these calculations indicated that a sample of 600 patients would provide an 8.1%-wide 95% confidence interval (95% CI) for the PPV estimate. Accordingly, 600 was deemed the minimum required sample size. However, because we expected more severe patients to be underrepresented in each health system’s population, instead of using random sampling, we disproportionately selected 75% of the target sample from patients who met “enriching” criteria focused on selected diagnoses, procedures, and/or medications that are known to be proxies for moderate-to-severe disease and relatively high levels of pain (Supplementary Materials, Additional File 1, Table 1). The remaining 25% of the sample was drawn from patients who had evidence of OA of the hip/knee but did not meet these “enriching” criteria. This weighted selection process was necessary to ensure that there was sufficient variability across disease severity to train and generate the ML-based algorithms.

**Table 1:** Clinical characteristics and the use of OA-related medications and procedures by patient type using claims data

Characteristics, N (%)	Patients without OA ^a^ (*N* = 47)	Case Patients^a^ (*N* = 360)	Comparator Patients^a^ (*N* = 83)
**Obesity**^b^	4 (8.5)	101 (28.1)	16 (19.3)
**Pain-related conditions**			
Joint pain of hip	2 (4.3)	46 (12.8)	5 (6.0)
Joint pain of knee	0 (0.0)	83 (23.1)	13 (15.7)
Joint pain in other site(s)	15 (31.9)	148 (41.1)	29 (34.9)
Back pain	0 (0.0)	10 (2.8)	2 (2.4)
Unspecified pain	12 (25.5)	168 (46.7)	19 (22.9)
**Other comorbidities**			
Hypertension	11 (23.4)	153 (42.5)	27 (32.5)
Diabetes	12 (25.5)	47 (13.1)	12 (14.5)
Gastroesophageal reflux	4 (8.5)	90 (25.0)	9 (10.8)
Depression	3 (6.4)	56 (15.6)	8 (9.6)
Anxiety	4 (8.5)	47 (13.1)	8 (9.6)
Drug or alcohol abuse	0 (0.0)	16 (4.4)	3 (3.6)
Insomnia	0 (0.0)	14 (3.9)	4 (4.8)
**Charlson Comorbidity Index category**			
0	32 (68.1)	230 (63.9)	58 (69.9)
1	9 (19.1)	64 (17.8)	14 (16.9)
2	1 (2.1)	26 (7.2)	4 (4.8)
≥ 3	5 (10.6)	40 (11.1)	7 (8.4)
**Medication use**			
Any NSAIDs	7 (14.9)	148 (41.1)	22 (26.5)
Topical NSAIDs	0 (0.0)	37 (10.3)	7 (8.4)
COX-2	0 (0.0)	37 (10.3)	7 (8.4)
Non-selective NSAIDs	7 (14.9)	117 (32.5)	17 (20.5)
Any opioids	13 (27.7)	178 (49.4)	20 (24.1)
Short-acting opioids	7 (14.9)	145 (40.3)	15 (18.1)
Long-acting opioids	0 (0.0)	4 (1.1)	1 (1.2)
Opioid mixed mechanism^b^	7 (14.9)	66 (18.3)	7 (8.4)
Oral corticosteroids	0 (0.0)	1 (0.3)	(0.0)
H_2_ blockers	3 (6.4)	33 (9.2)	9 (10.8)
**IA steroids injections**	10 (21.3)	288 (80.0)	21 (25.3)
**HA injections**	1 (2.1)	89 (24.7)	3 (3.6)
**OA related Surgeries**			
Hip replacement	0 (0.0)	80 (22.2)	2 (2.4)
Knee replacement	1 (2.1)	134 (37.2)	4 (4.8)
**Nerve Block Ablation**	7 (14.9)	208 (57.8)	14 (16.9)

^a^Based on a total of 490 patients who had available claims data. Non-OA group refers to patients without evidence of OA of hip or knee; OA case group refers to patients with all three OA features – OA of hip/knee, moderate-to-severe disease and inadequate response to two or more pain-related medications; comparator patients are all other patients with OA of hip/knee

^b^Defined using relevant diagnosis and procedure codes, and body mass index

^‡^Including tapentadol and tramadol

Abbreviations: *COX-2*  Cyclooxygenase-2, *OA* Osteoarthritis, *HA*  Hyaluronic acid, *IA*  Intraarticular, *NSAIDs* Nonsteroidal anti-inflammatory drugs

## Data extraction period

The data extraction period for each patient in the study sample was defined to include the most recent 18-month period available (i.e., extracted data reflected most recent treatment patterns and experiences with OA and of current standards of care at the time the study was initiated). The end of the 18-month extraction period was defined as the earliest of: (1) knee or hip arthroplasty (including total and partial arthroplasty, where applicable); (2) end date of the patient’s insurance enrollment period; or (3) end of the study period. The most recent knee or hip arthroplasty was selected as a potential terminus for the data extraction period because the procedure is expected to alleviate disease to the extent that subsequent to recovery, patients are no longer considered to have moderate-to-severe OA (and therefore no longer require analgesics to alleviate OA pain) in the affected joint. Information on diagnosis and severity of OA, pain assessments, imaging, diagnostic tests, and physicians’ notes on pain management during the extraction period were abstracted from patients’ medical charts.

## Chart review

At least two authors (AB, MS, or MLG) independently reviewed abstracted information from medical charts for each patient and adjudicated the presence or absence of the three relevant OA-related characteristics (i.e., OA of hip and/or knee, moderate-to-severe disease, inadequate response to at least two pain-related medications). Decision rules used for the chart review are shown in the Supplementary Materials (Additional File 1, Table 2). Disagreements between reviewers were resolved through adjudication by the study clinician (CTH). The group assignment based on the chart review (i.e., whether a patient had each of the characteristics of interest) served as the “gold standard” for assessing the performance of predefined and ML-based algorithms (i.e., performance in assigning patients into the correct categories).

**Table 2:** Performance of predefined algorithms to identify patients with OA of hip and/or knee (Step 1, total *N* = 490 with available claims data)

Algorithm	TP	FP	TN	FN	Sensitivity	Specificity	NPV	PPV	Accuracy
1. ≥ 2 medical (inpatient and/or outpatient [both physicians’ office visits and all other outpatient settings]) encounters resulting in diagnoses OA of the hip and/or knee12 within 6 months	350	7	40	93	0.79	0.85	0.30	0.98	0.80
2. ≥ 2 medical encounters resulting in diagnoses of OA of the hip and/or knee ≤ 90 days apart	335	6	41	108	0.76	0.87	0.28	0.98	0.77
3. 1 inpatient admission resulting in a diagnosis of OA of the hip and/or knee	239	2	45	204	0.54	0.96	0.18	0.99	0.58
4. A diagnosis of OA of the hip and/or knee from ≥ 1 outpatient visit	401	21	26	42	0.91	0.55	0.38	0.95	0.87
5. A diagnosis of OA of the hip and/or knee from ≥ 2 outpatient visits in the same CY	345	6	41	98	0.78	0.87	0.29	0.98	0.79
6. A diagnosis of OA of the hip and/or knee from ≥ 3 outpatient visits in the same CY	294	6	41	149	0.66	0.87	0.22	0.98	0.68
7. A diagnosis of OA of the hip and/or knee from ≥ 4 outpatient visits in the same CY	263	2	45	180	0.59	0.96	0.20	0.99	0.63
8. A diagnosis of OA of the hip and/or knee from ≥ 1 outpatient visit; and ≥ X-rays of the hip and/or knee in the same CY	351	10	37	92	0.79	0.79	0.29	0.97	0.79
9. A diagnosis of OA of the hip and/or knee from ≥ 2 outpatient visits; and ≥ X-rays of the hip and/or knee in the same CY	318	3	44	125	0.72	0.94	0.26	0.99	0.74
10. A diagnosis of OA of the hip and/or knee from ≥ 3 outpatient visits; and ≥ X-rays of the hip and/or knee in the same CY	270	3	44	173	0.61	0.94	0.20	0.99	0.64
11. A diagnosis of OA of the hip and/or knee from ≥ 4 outpatient visits; and ≥ X-rays of the hip and/or knee in the same CY	244	1	46	199	0.55	0.98	0.19	1.00	0.59
12. 1 outpatient visit and ≥ 2 diagnoses of OA of the hip and/or knee from outpatient visits within 5 years	274	5	42	169	0.62	0.89	0.20	0.98	0.64
13. ≥ 2 outpatient visits resulting in diagnoses of OA of the hip and/or knee within 5 years	351	6	41	92	0.79	0.87	0.31	0.98	0.80
14. ≥ 2 outpatient visits resulting in diagnoses of OA of the hip and/or knee within 2 years	351	6	41	92	0.79	0.87	0.31	0.98	0.80
15. ≥ 1 medical encounter resulting in a diagnosis of OA of the hip and/or knee	401	21	26	42	0.91	0.55	0.38	0.95	0.87
16. ≥ 1 medical encounter resulting in a diagnosis of OA of the hip or knee plus ≥ 1 medical encounter resulting in a diagnosis of joint pain ≥ 30 days apart	325	4	43	118	0.73	0.91	0.27	0.99	0.75
17. ≥ 1 medical encounter resulting in a diagnosis of OA of the hip or knee plus ≥ 1 administration of either HA or IA corticosteroids	316	8	39	127	0.71	0.83	0.23	0.98	0.72
18. ≥ 1 medical encounter resulting in a diagnosis of OA of the hip or knee plus ≥ 1 relevant scans (i.e., X-ray, MRI) of knee or hip ± 2 years of the OA diagnosis	358	7	40	85	0.81	0.85	0.32	0.98	0.81
19. ≥ 1 medical encounter resulting in a diagnosis of OA of the hip or knee plus ≥ 1 procedure codes for knee or hip arthroplasty (partial or full; initial or revision) within 30 days after OA diagnosis	231	0	47	212	0.52	1.00	0.18	1.00	0.57
20. ≥ 1 medical encounter resulting in a diagnosis of OA of the hip or knee plus ≥ 1 mobility aid (e.g., cane, walker, wheelchair) ± 30 days of OA diagnosis	79	2	45	364	0.18	0.96	0.11	0.98	0.25
**Combined Algorithm (using OR function)**									
Combinations of all algorithms with performance ≥ 0.98 PPV	382	11	36	61	0.86	0.77	0.37	0.97	0.85
Combinations of all algorithms with performance ≥ 0.95 PPV	403	21	26	40	0.91	0.55	0.39	0.95	0.88
Combinations of all algorithms with performance ≥ 0.90 PPV	403	21	26	40	0.91	0.55	0.39	0.95	0.88
Combinations of all algorithms with performance ≥ 0.85 PPV	403	21	26	40	0.91	0.55	0.39	0.95	0.88
Combinations of all algorithms with performance ≥ 0.80 PPV	403	21	26	40	0.91	0.55	0.39	0.95	0.88

Abbreviations: *COX-2* cyclooxygenase-2, *CY* Calendar year, *FN* False negative, *FP* False positive, *HA *Hyaluronic acid, *IA *Intraarticular, *MRI *Magnetic resonance imaging, *NPV *Negative predictive value, *NSAIDs *Nonsteroidal anti-inflammatory drugs, *OA* Osteoarthritis, *PPV *Positive predictive value, *TN *True negative, *TP *True positive

### Study variables

Several measures taken from patients’ electronic data and charts were used to develop and refine the case-identification algorithms. Demographic characteristics included age, gender, race/ethnicity, weight, height, and body mass index (BMI). Clinical characteristics, which were identified using ICD-9-CM or ICD-10-CM diagnosis codes, included unique comorbidities (chronic conditions, other sources of joint pain, and unspecific pain) and Quan’s version of the Charlson Comorbidity Index [12]. To avoid potential misclassification, comorbidities were established based on the presence of two or more outpatient encounters at least 30 days apart, or one inpatient encounter. Common pharmacological and non-pharmacological treatments used for OA were also identified using National Drug Codes (NDC) and procedure codes (in ICD-9-CM, ICD-10-CM, Current Procedural Terminology 4th edition, and/or Healthcare Common Procedures Coding System formats) as relevant. Pharmacotherapy included nonsteroidal anti-inflammatory drugs (NSAIDs), opioids (short- and long-acting and mixed mechanism), corticosteroids (including intraarticular [IA] injections), and hyaluronic acid (HA) injections. Key non-pharmacological treatments included, but were not limited to, hip and/or knee arthroplasty (partial or full), and nerve blocks/ablation. In addition, use of healthcare services (inpatient hospital admissions, emergency department visits, outpatient physicians’ office visits, other outpatient visits, and use of durable medical equipment) was assessed. Costs of OA-related pharmacological treatments and OA-specific healthcare services (defined as medical encounters resulting in a diagnosis of OA of hip/knee) were estimated using reimbursed amounts as identified in claims data. 

### Algorithm development

#### Predefined algorithms

A targeted literature review was performed to develop predefined case-identification algorithms. The literature search was conducted using EMBASE for publications from 1 January 2004 to 13 August 2019, supplemented by searching relevant conference publications and guidelines from OA- and pain-specific organizations (i.e., Osteoarthritis Research Society International [OARSI], Osteoarthritis Foundation International [OAFI], Arthritis National Research Foundation [ANRF], American Academy of Orthopedic Surgeons [AAOS], American College of Rheumatology [ACR], American Pain Society [APS], International Association for the Study of Pain [IASP], World Health Organization [WHO]). Results from this review, augmented by input from clinical experts (CTH, BJ), informed the development of a set of algorithms designed to identify OA of hip and/or knee, moderate-to-severe OA, and inadequate response to pain medications. 

#### Machine learning-derived algorithms

In addition to the predefined algorithms, we also applied three supervised ML methods (i.e., logistic regression, classification, and regression tree [CART], random forest [RF]) to derive algorithms that could identify patients with all three OA characteristics (i.e., moderate-to-severe OA of the hip and/or knee with inadequate response to at least two pain-related medications). These methods, which were adapted for use with longitudinal data, were applied separately to the claims and EMR data, because different data elements were available in each type of data source and because, even for information that was available in both types of data, some elements were recorded differently [13]. As previous research has shown that algorithms already exist that are able to identify patients with OA with high accuracy, [7–9] we designed the ML analyses to focus on ascertainment of patients with moderate-to-severe OA (vs. mild OA) and on patients with inadequate response to pain-related therapies. Consequently, our ML analyses excluded patients without evidence of OA.

A nested cross-validation procedure was used to evaluate the ML models resulting from both the claims and EMR analyses. This procedure divides the data into a series of training, validation, and testing sets. It trains and selects hyperparameters in the inner loop and estimates the generalization error in the outer loop. Nested cross-validation, which is well-suited for relatively small samples, is an effective approach that allows each patient to be used for training and validating the hyperparameters. Since there were more case than comparator patients, we applied stratified random sampling with replacement on the training dataset, using the Synthetic Minority Oversampling Technique (SMOTE), [14] to create a more balanced synthetic training data set. The RF method with recursive feature elimination was used to select the best features in each model (i.e., claims and EMR models). Different thresholds (ranging from 20 to 50) were tested and implemented based on the number of features obtained using RF (wrapping method) for features selection. Nested cross-validation approaches were used for both the claims and EMR datasets due to the relatively small sample sizes. Parameter tuning methods were also used to obtain the best configuration of parameters for each ML method. Details are provided in the Supplemental Materials (Additional File 2).

### Algorithm assessment

We assessed the performance of all algorithms by comparing, for each patient, group assignment (case or comparator) of predefined and ML-based algorithms against the benchmark assignment obtained from the chart review. Each algorithm-based group assignment was classified as: true positive (TP) (i.e., assigned by an algorithm as having the characteristic[s] of interest and confirmed by the chart review), false positive (FP) (i.e., assigned by an algorithm as having the characteristic[s] of interest but not so by the chart review), true negative (TN) (i.e., assigned by an algorithm as not having the characteristic[s] of interest and confirmed by the chart review), or false negative (FN) (i.e., assigned by an algorithm as not having the characteristic[s] of interest but not so by the chart review). We used five performance metrics to assess each algorithm: sensitivity, specificity, negative predictive value (NPV), PPV, and accuracy.

We assessed the performance of the individual predefined algorithms and of combinations of high-performing predefined algorithms. Briefly, the latter involved first combining individual, high-performance algorithms (e.g., with a PPV of 98% or higher) using the OR function, meaning that if any of these individual algorithms were satisfied, the patient was considered as having the OA characteristic of interest. If none of the algorithms classified the patient as having the OA characteristic of interest, then the patient was considered as not having the OA characteristic of interest. The classification results were subsequently compared against the chart review classification; performance metrics for the combined algorithms were recalculated based on the updated TP, TN, FP, and FN values.

We additionally reported the area under the receiver-operator curve and the F1 score of ML-derived algorithms (definitions of these metrics are presented in the Supplementary Materials, Additional File 2) as well as mean absolute Shapley Additive exPlanations (SHAP) values for the features included in the final model. The SHAP values quantify how much impact a particular feature makes toward predicting the target class (i.e., case vs. comparator) with positive and negative SHAP values signaling positive and negative contributions, respectively, to the target class membership. 

Data management and descriptive analyses were performed using SAS® version 9.4 and the ML tasks were performed using Python version 3.9 using the scikit-learn library (the Pandas, NumPy, and SciPy libraries were used for data preprocessing and analysis). Although we performed the ML analyses using claims and EMR data, we focus our presentation here on the claims-based results because the value added by our trained ML algorithms is likely to be greater for claims than EMR data (EMR-based results are presented in the Supplementary Materials, Additional File 1).

## Results

### Patient characteristics

**Fig. 1:**
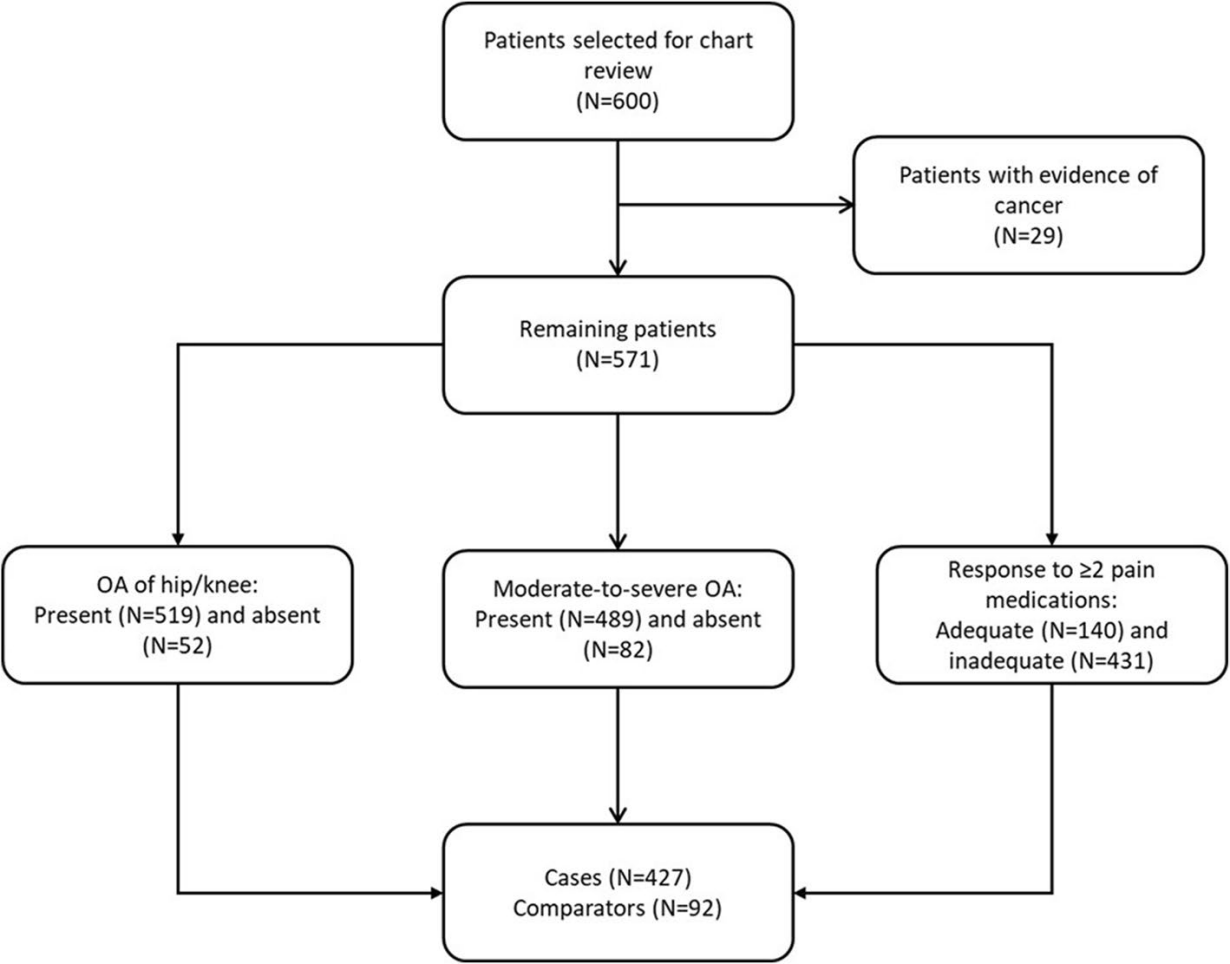
Study sample selection and attrition. Abbreviation OA = osteoarthritis

After applying the selection criteria to the claims and EMR data, 29 patients had some evidence of cancer and were subsequently excluded (Fig. 1). The final study sample, therefore, included 571 patients, of whom 483 (84.6%) had both claims and EMR data available, 81 (14.1%) had only EMR data available, and 7 (1.2%) had only claims data available. Based on the chart review, 519 (90.9%) patients had OA of hip and/or knee, 489 (85.6%) had moderate-to-severe OA, and 431 (75.5%) had inadequate response to two or more pain-related medications. Out of the 519 patients with OA of hip and/or knee, 427 had all three OA-related characteristics and served as cases in the ML analyses (the other 92 patients served as comparators).

Patients with OA of hip/knee were slightly older than those without (mean age: 63.2 vs. 60.6 years) and were nominally more likely to be women (64.4% vs. 63.8%). Most patients were White (70.0% and 53.2% of those with and without OA of hip/knee, respectively). Clinical characteristics based on claims data, stratified by patient type, are summarized in Table 1. A larger percentage of case patients had evidence of obesity than comparator patients and patients without OA (28.1% vs. 19.3% and 8.5%, respectively). Pain-related conditions were more frequently recorded among case patients than other patients. Diagnoses of joint pain of the hip and the knee were recorded in 12.8% and 23.1% of case patients, respectively. Common comorbidities included hypertension, diabetes, gastroesophageal reflux, depression, and anxiety; other comorbidities included drug or alcohol use disorders and insomnia. Comorbidities, apart from diabetes, were more likely to be identified among patients with OA (including case and comparator patients) than those without OA. A higher proportion of case patients used NSAIDs and opioids than comparator patients (Table 1). Non-selective NSAIDs and short-acting opioids were among the most commonly used pain-related medications (by 32.5% and 40.3% of case patients, by 20.5% and 18.1% of comparator patients, and 14.9% and 14.9% of patients without OA). Hip and knee surgeries were performed on 22.2% and 37.2% of case patients, respectively and 2.4% and 4.8% of comparator patients, respectively. Nerve block/ablation was performed on 57.8% of case patients and 16.9% of comparator patients, respectively.

## Performance of predefined algorithms

### Algorithms for identifying OA of hip and/or knee

Each predefined algorithm developed to identify OA of hip/knee resulted in PPV in the range 0.95–1.00 and specificity values in the range 0.55–1.00 (Table 2). All but one algorithm (#20), had sensitivity values ≥ 0.52 and accuracy values ≥ 0.57. However, all algorithms had relatively poor NPV (range: 0.11–0.38). Two algorithms (#4: “diagnosis of OA of the hip and/or knee from ≥ 1 outpatient visits” and #15: “ ≥ 1 medical encounters resulting in a diagnosis of OA of the hip and/or knee”) had the same performance and the greatest accuracy (0.87) and sensitivity (0.91) (PPV: 0.95, NPV: 0.38). Performance improved slightly when combining high-performing algorithms into one algorithm. For example, combining all algorithms with PPV ≥ 0.95 resulted in group classification that achieved relatively high sensitivity (0.91), PPV (0.95), and accuracy (0.88) but low specificity (0.55) and NPV (0.39).

### Algorithms for identifying moderate-to-severe OA

Algorithms for identifying moderate-to-severe disease status had high specificity (range: 0.82–1.00) and PPV (range: 0.83–1.00) (Table 3). However, sensitivity and NPV values varied substantially across these algorithms, ranging from 0.00 to 0.80 and from 0.16 to 0.47, respectively. The algorithm with the greatest accuracy (0.83) and sensitivity (0.80) was #7: “ ≥ 1 administrations of HA or IA corticosteroids” (specificity: 0.96, PPV: 0.99, NPV: 0.47) that identified 331/413 patients with moderate-to-severe OA of the hip/knee. FNs among positive cases (i.e., FNs/[FNs + TPs]) ranged from 0.54 to 1.00. When all algorithms with PPV ≥ 0.95 were combined, sensitivity, specificity, NPV, PPV and accuracy was 0.90, 0.83, 0.61, 0.97 and 0.89, respectively.

**Table 3:** Performance of predefined algorithms to identify patients with moderate-to-severe OA of the hip and/or knee (Step 2, total *N* = 490 with available claims data)

Algorithm	TP	FP	TN	FN	Sensitivity	Specificity	NPV	PPV	Accuracy
1. ≥ 1 code for any OA-related surgical procedures	263	0	77	150	0.64	1.00	0.34	1.00	0.69
2. ≥ 2 medical encounters on different days resulting in diagnosis of a relevant psychiatric comorbidity (i.e., anxiety, depression), with at least two encounters for psychiatric comorbidities both occurring within 180 days of any OA diagnosis	87	14	63	326	0.21	0.82	0.16	0.86	0.31
3. ≥ 2 medical encounters resulting in diagnosis of relevant psychiatric comorbidities, with at least two encounters for the psychiatric comorbidities within 90 days of any OA diagnosis	83	12	65	330	0.20	0.84	0.16	0.87	0.30
4. ≥ 2 prescriptions for opioids within 90 days of any OA diagnosis	66	5	72	347	0.16	0.94	0.17	0.93	0.28
5. ≥ 2 prescriptions for oral NSAIDs (including COX-2 inhibitors) within 90 days of any OA diagnosis	1	0	77	412	0.00	1.00	0.16	1.00	0.16
6. ≥ 2 prescriptions for topical NSAIDs (including COX-2 inhibitors) within 90 days of any OA diagnosis	10	2	75	403	0.02	0.97	0.16	0.83	0.17
7. ≥ 1 administration of HA or IA corticosteroids	331	3	74	82	0.80	0.96	0.47	0.99	0.83
8. ≥ 2 administrations of HA or IA corticosteroids at least 90 days apart	191	1	76	222	0.46	0.99	0.26	0.99	0.54
9. ≥ 1 administration of a nerve block ± 30 days of any OA diagnosis	191	3	74	222	0.46	0.96	0.25	0.98	0.54
10. ≥ 1 claim for a mobility aid including walking cane, walker, and wheelchair, or crutch ± 30 days of any OA diagnosis	79	5	72	334	0.19	0.94	0.18	0.94	0.31
11. ≥ 1 code for physical and/or occupational therapy ± 30 days of any OA diagnosis	175	12	65	238	0.42	0.84	0.21	0.94	0.49
12. OA-related costs > 30% mean OA-related costs for the cohort	147	0	77	266	0.36	1.00	0.22	1.00	0.46
13. OA-related costs > 50% mean OA-related costs for the cohort	0	0	77	413	0.00	1.00	0.16	N/A	0.16
14. ≥ 2 codes for X-ray examinations in a 1-year period	295	10	67	118	0.71	0.87	0.36	0.97	0.74
**Combined algorithm (using OR function)**									
Combinations of all algorithms with performance ≥ 0.98 PPV	360	6	71	53	0.87	0.92	0.57	0.98	0.88
Combinations of all algorithms with performance ≥ 0.95 PPV	372	13	64	41	0.90	0.83	0.61	0.97	0.89
Combinations of all algorithms with performance ≥ 0.90 PPV	378	21	56	35	0.92	0.73	0.62	0.95	0.89
Combinations of all algorithms with performance ≥ 0.85 PPV	378	25	52	35	0.92	0.68	0.60	0.94	0.88
Combinations of all algorithms with performance ≥ 0.80 PPV	378	26	51	35	0.92	0.66	0.59	0.94	0.88

Abbreviations: *COX-2 *Cyclooxygenase-2, *FN *False negative, *FP *False positive, *HA *Hyaluronic acid, *IA *Intraarticular, *NPV *Negative predictive value, *NSAIDs *Nonsteroidal anti-inflammatory drugs, *OA *Osteoarthritis, *PPV *Positive predictive value, *TN *True negative, *TP *True positive

### Algorithms for identifying inadequate response to two or more pain medications

The best-performing algorithm (#3) for identifying inadequate response to at least two pain medications was “receipt of knee or hip arthroplasty (partial or complete; original or revision)” (sensitivity: 0.71, specificity: 0.95, NPV: 0.54, PPV: 0.98, accuracy: 0.77) (Table 4), followed by algorithm #4: “receipt of nerve block” (sensitivity: 0.58, specificity: 0.85, NPV: 0.41, PPV: 0.92, accuracy: 0.65). The other four algorithms failed to identify the majority of patients with inadequate response to their pain medications (FNs between 90 to 98%). After combining all algorithms with PPV ≥ 0.90, the FN rate was 20% (sensitivity: 0.80; specificity: 0.82; NPV: 0.59, PPV: 0.93, accuracy: 0.80).

**Table 4:** Performance of predefined algorithms to identify patients with inadequate response to two or more pain-related medications (Step 3, total *N* = 490 with available claims data)

Algorithm	TP	FP	TN	FN	Sensitivity	Specificity	NPV	PPV	Accuracy
1. Receipt of HA, IA corticosteroid, and/or nerve block preceded by receipt of 2 different classes of analgesics (i.e., NSAIDs [including COX-2 inhibitors], opioids)	10	0	127	353	0.03	1.00	0.26	1.00	0.28
2. Receipt of ≥ 3 analgesic classes	44	1	126	319	0.12	0.99	0.28	0.98	0.35
3. Receipt of knee or hip arthroplasty (partial or complete; original or revision)	258	6	121	105	0.71	0.95	0.54	0.98	0.77
4. Receipt of nerve block	210	19	108	153	0.58	0.85	0.41	0.92	0.65
5. Receipt of ≥ 2 different opioids within a 90-day period	24	4	123	339	0.07	0.97	0.27	0.86	0.30
6. Receipt of a mobility aid (as described above) within 90-day period subsequent to receipt of an opioid	12	0	127	351	0.03	1.00	0.27	1.00	0.28
**Combined algorithm (using OR function)**									
Combinations of all algorithms with performance ≥ 0.98 PPV	21	0	127	342	0.06	1.00	0.27	1.00	0.30
Combinations of all algorithms with performance ≥ 0.95 PPV	262	6	121	101	0.72	0.95	0.55	0.98	0.78
Combinations of all algorithms with performance ≥ 0.90 PPV	290	23	104	73	0.80	0.82	0.59	0.93	0.80
Combinations of all algorithms with performance ≥ 0.85 PPV	291	25	102	72	0.80	0.80	0.59	0.92	0.80
Combinations of all algorithms with performance ≥ 0.80 PPV	291	25	102	72	0.80	0.80	0.59	0.92	0.80

Abbreviations: *COX-2 *Cyclooxygenase-2, *FN *False negative, *FP *False positive, *HA *Hyaluronic acid, *IA *Intraarticular, *NPV *Negative predictive value, *NSAIDs *Nonsteroidal anti-inflammatory drugs, OA Osteoarthritis, *PPV *Positive predictive value, *TN *True negative, *TP *True positive

### Algorithms for identifying patients with all three characteristics

Combining predefined algorithms (i.e., algorithms for OA of hip and/or knee with PPV ≥ 0.95, algorithms for moderate-to-severe OA with PPV ≥ 0.95, and algorithms for inadequate response with PPV ≥ 0.90) resulted in identification of patients with all three relevant features with a sensitivity of 0.95, specificity of 0.26, NPV of 0.65, PPV of 0.78, and overall accuracy of 0.77.

### Performance of ML-based algorithms, claims data

**Fig. 2:**
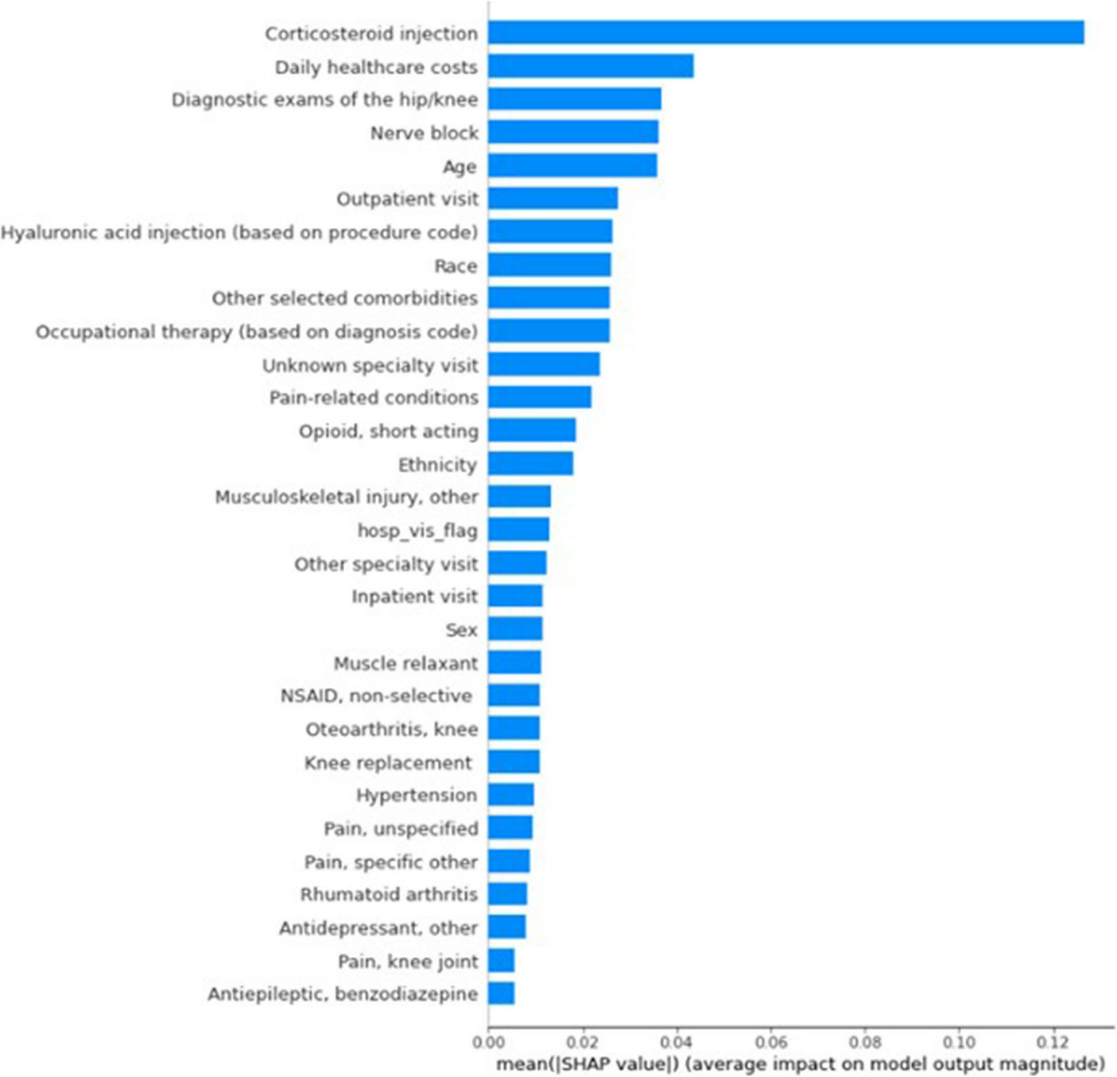
Feature of importance ranking based on mean absolute Shapley Additive exPlanations (SHAP) Values, from random forest model using claims data

Table 5 displays the performance metrics for the ML algorithms that were trained using claims data. Although all three methods performed well, with overall PPV ranging from 0.88 to 0.92, NPV ranging from 0.47 to 0.62, and accuracy ranging from 0.75 to 0.83, the RF method resulted in the best performance across relevant metrics (the logistic regression and CART methods resulted in very similar values for these metrics). The performance metrics for the EMR-based ML algorithms were similar and are shown in the Supplementary Materials (Additional File 1, Table 3). Figure 2 shows the features included in the final model in descending order of importance based on the mean SHAP values. The five most important features (and their mean absolute SHAP values) were having had a corticosteroid injection (0.127), daily health care costs (0.044), having undergone diagnostic examinations of hip/knee (0.037), having had a nerve block procedure (0.036), and age (0.036). The corresponding SHAP forest plots are provided in the Supplementary Materials (Additional File 1, Fig. 1). SHAP figures and list of features derived from the EMR data are shown in the Supplementary Materials (Additional File 1). Binary versions of the trained models (i.e., Python pickle files) and associated Jupyter notebook files for executing these models are provided in the Supplementary Materials (Additional Files 3–8).

**Table 5:** Performance of machine learning algorithms to identify patients with moderate-to-severe OA of the hip and/or knee with inadequate response to two or more pain-related medications applied to claims data

Performance Metric	Logistic Regression	Classification and Regression Tree	Random Forest
Positive Predictive Value (SD)^a^	0.88 (0.02)	0.89 (0.03)	0.92 (0.02)
Negative Predictive Value	0.47	0.48	0.62
Sensitivity	0.77	0.78	0.86
Specificity	0.66	0.67	0.75
Accuracy	0.75	0.75	0.83
Area Under the Curve	0.72	0.73	0.81
F1^b^	0.82	0.83	0.89

^a^The mean and SD are estimated from the outer folds of the nested cross validation

^b^F1 score is calculated as the weighted average of PPV and sensitivity. A value between 0 and 1 with 1 being the highest (most accurate)

Abbreviations: *OA*  Osteoarthritis, *SD*  Standard deviation

## Discussion

The purpose of the this study was to develop and validate algorithms that can be used to identify patients with moderate-to-severe OA of hip/knee and inadequate response to at least two pain medications using structured claims or EMR data. The individual predefined algorithms based on the literature and expert opinion performed well in identifying patients with the three sets of individual characteristics when measured by PPV. However, predefined algorithms were less accurate for identifying moderate-to-severe disease and, especially those for identifying inadequate response to at least two pain medications. The predefined algorithms were also characterized by low sensitivity and NPV, meaning that although these algorithms could identify a good proportion of patients with characteristics of interest, they were less able to identify patients without these characteristics (i.e., high numbers of FPs). The combined predefined algorithm for identifying patients with all three OA characteristics performed well in identifying patients with OA but, like their component algorithms, had fairly low specificity and NPV. Accordingly, while the predefined algorithms have good face validity, are transparent, and are easy to understand, they also misclassify large portions of patients of interest as evidenced by relatively low sensitivity and NPV and the large proportions of FNs. This misclassification is most likely due to the fact that the predefined algorithms ignore other aspects of patients’ medical history and treatment patterns and because were not developed by training them in the presence of gold-standard data. 

All three ML methods performed better than the combined predefined algorithm for differentiating between case and comparator patients. Furthermore, we found that the RF method was the best performing one regardless of the source of data. Although the logistic regression method did not perform as well as the RF method when applied to either source of data, it is the most transparent and easiest ML method to implement and modify by other researchers. The RF and CART methods, on the other hand, are less transparent and more difficult for other researchers to use and modify, but they are, as applied to the data used in this study, the best performing.

The ability to efficiently identify patients with OA and to identify the severity of disease (and pain) and efficacy of medications will allow for more efficient and effective disease management. Use of algorithms or ML techniques can also help researchers to identify and stratify patients in clinical trials of novel therapies for OA [15]. The descriptions of these methods, their results, and the trained models are being shared to promote their use and to foster transparency[16]. We believe that sharing these methods and results can lead to more effective and efficient algorithms in the future, which in turn, can be used to improve research and patient care. This approach is not limited to OA, as the ML techniques presented here can be adapted for other medical conditions as well. 

The performance of predefined algorithms in identifying patients with hip/knee OA has been reported in previous studies, with similarly high PPVs as reported here [7–9]. However, this is the first study, to our knowledge, to develop algorithms that can differentiate between patients with moderate-to-severe OA and inadequate response to at least two pain medications and other patients with OA of the hip/knee. Although EMR data generally contain richer information on clinical and demographic characteristics than claims data, we focused on claims because of the value these algorithms can add to data that lack rich clinical information, and because these algorithms can not only be used to identify patients of interest and their treatment journeys, but also because they can enable economic analyses based on claims data. 

The results of these analyses should be considered alongside limitations inherent to claims and EMR data. The most important limitation is potentially incomplete and/or incorrect data. The degree to which data are incomplete, or missing, or incorrectly entered into EMR databases is highly dependent on clinician-level factors such as workflow, patient volume, and familiarity with the particular EMR system. Furthermore, even though the data were obtained from IDNs, patients could have received healthcare outside of those systems and any such activities or encounters were not captured. In addition, like other analyses of administrative data, we were unable to determine if patients consumed their filled prescriptions or if other therapies were optimized when some medications were discontinued. Another limitation is the relatively short data extraction period. Chart review is a time-consuming and expensive process; consequently, only data from the most recent 18 months within the study period were abstracted. Furthermore, it is challenging to distinguish inadequate response versus intolerance to pain-related medications in database studies because reasons for medication change are typically not recorded in charts or EMR data. Similarly, pain scores as recorded in charts may not reflect pain exclusive to the knees or hip, as a large proportion of patients with OA also have evidence of pain, especially self-reported pain, in other parts of the body [17] and because the site(s) for which pain was assessed were often not documented. Lastly, although the current study demonstrated that the application of algorithms can accurately assign patients into appropriate patient subgroups, further study is necessary to predict the timepoint of change in disease severity status (e.g., the incidence date of moderate-to-severe OA), which is often challenging to capture within electronic healthcare data. We used data from two IDNs to reduce bias caused by variations in patient characteristics and OA treatment pathways. Further study is needed to repeat the methods in other systems to assess the performance of ML-based algorithms. 

## Conclusions

Although predefined algorithms performed adequately in identifying each OA characteristic of interest (i.e., presence of OA of hip/knee, moderate-to-severe disease, or inadequate response to pain medications), more sophisticated algorithms using ML-based methods better differentiated between levels of disease severity and whether patients adequately respond to at least two pain medications. Further work is needed to better understand the specific relationship(s) between important features (predictors) identified and disease severity/adequacy of pain management. This study demonstrates that ML can be used to further unlock the utility of claims and/or EMR data in examining this important — and currently underserved — population of patients with OA of the hip/knee as well as in the application of these methods to other disease states.

## Supplementary Information

**Additional file 1.** Supplementary Results Tables and Figures.

**Additional file 2.** Supplementary Information on Machine Learning Methods.

**Additional file 3.** Final Trained ML Models (Pickle File), Claims Data.

**Additional file 4.** Final Predictive Features (Pickle File), Claims Data.

**Additional file 5.** Jupyter Notebook for Executing the Trained Models from Claims Data.

**Additional file 6.** Final Trained ML Models (Pickle File), EMR Data.

**Additional file 7.** Final Predictive Features (Pickle File), EMR Data.

**Additional file 8.** Jupyter Notebook for Executing the Trained Models from EMR Data.

## Data Availability

The raw data that support the findings of this study are available from Henry Ford Health System and Reliant Medical group, respectively. Restrictions apply to the availability of these data, which were used under license for the current study, and so are not publicly available. Materials that are generated from the current study are included in this published article and its supplementary information files.

## Abbreviations

AAOS: American Academy of Orthopedic Surgeons
APS: American Pain Society
ACR: American College of Rheumatology
ANRF: Arthritis National Research Foundation
BMI: Body mass index
CART: Classification and regression tree
CI: Confidence interval
EMR: Electronic medical records
FN: False negative
FP: False positive
H_2_: Histamine 2
HA: Hyaluronic acid
HAP: Health Alliance Plan
HFHS: Henry Ford Health System
IA: Intraarticular
IASP: International Association for the Study of Pain
ICD-9-CM: International Classification of Disease Version 9 Clinical Modification
ICD-10-CM: International Classification of Disease Version 10 Clinical Modification
IDN: Integrated delivery networks
IRB: Institutional Review Board
ML: Machine learning
MRI: Magnetic resonance imaging
NDC: National Drug Codes
NPV: Negative predictive value
NSAID: Nonsteroidal anti-inflammatory drug
OA: Osteoarthritis
OAFI: Osteoarthritis Foundation International
OARSI: Osteoarthritis Research Society International
PPVs: Positive predictive values
RF: Random forest
SHAP: Shapley Additive exPlanations
SMOTE: Synthetic Minority Oversampling TEchnique
TN: True negative
TP: True positive
UI: Uncertainty interval
US: United States
WHO: World Health Organization
