# Supplementary material for: Use of electronic health data to identify patients with moderate-to-severe osteoarthritis of the hip and/or knee and inadequate response to pain medications
Source: BMC Med Res Methodol. 2023 Jun 30;23:156. doi: 10.1186/s12874-023-01964-y (PMC10311749; doi:10.1186/s12874-023-01964-y)
Supplement: Supplementary file 1 — Additional file 1. Supplementary Results Tables and Figures. [file 12874_2023_1964_MOESM1_ESM.docx]

**Supplementary Materials**

**Use of Electronic Health Data to Identify Patients with Moderate-to-Severe Osteoarthritis of the Hip and/or Knee and Inadequate Response to Pain Medications**

# Supplementary Materials

## Additional File 1. Supplementary Results Tables and Figures

**Supplemental Table 1. Criteria used to select patients with OA of the hip/knee, moderate-to-severe disease, and inadequate response to pain medications, respectively**

| **OA Characteristic / Criterion** |
| --- |
| Patients are considered to have OA of the hip/knee, if any of the following criteria is satisfied |
| 1 hospitalization* OR 2 physicians’ office visits* OR 1 physician office visit* and 2 prescriptions** in 5 years |
| 1 hospitalization* OR 2 physicians’ office visits* in 5 years |
| 2 physicians’ office visits* in 5 years |
| Patients are considered to have moderate-to-severe disease if any of the following criteria is satisfied |
| 1 administration of hyaluronic acid* |
| 1 administration of intraarticular corticosteroids* |
| Use of a cane, walker, or wheelchair |
| ≥6 physical/occupational therapy visits within a 12-month period |
| Patients are considered to have inadequate response to pain medications, if any of the following criteria is satisfied |
| ≥3 different classed of pain-related medications† |
| ≥4 pain scores ≥5 within a 12-month period |
| ≥1 nerve block injection |
| Hip or knee arthroplasty‡ |

Notes: *With relevant diagnosis of OA of hip/knee. **Defined as medications commonly used to treat nociceptive pain, including topical nonsteroidal anti-inflammatory drugs (NSAIDs), oral NSAIDs, duloxetine, and opioids. †Defined to include nonsteroidal anti-inflammatory drugs (NSAIDs), corticosteroids, hyaluronic acid, and opioids. ‡Hip or knee arthroplasty includes both partial and total arthroplasty but excludes revision of arthroplasty.

Abbreviation = OA, osteoarthritis

**Supplemental Table 2. Definition of rules for assessing OA characteristics in chart review data**

| **Element** | **Decision Rule(s)** | **Date when criterion was satisfied** |
| --- | --- | --- |
| **OA of hip and/or knee** | - Medical diagnosis of OA of hip/knee; ***OR*** - Radiology report confirming OA of hip/knee | - Earliest date with confirmatory evidence during extraction period was used as date criterion was satisfied |
| **Moderate-to-severe OA** | - ≥4 on a 0-10 pain scale due to OA; ***OR*** - Total or partial knee/hip joint replacement; ***OR*** - Radiologic evidence suggesting moderate-to-severe OA (e.g., radiologist notes, Kellgren-Lawrence grading score 3 or 4 or radiology report of degenerative changes consistent with this score, such as “moderate-to-severe joint space narrowing”); ***OR*** - Use of mobility aid due to OA, ***OR*** - Diagnosis of OA of hip/knee and either - >2 prescriptions for the same opioid-containing drug (including single ingredient and compound products) >1 week apart; ***OR*** - 1 IA steroid or HA injection | - Earliest date with confirmatory evidence during extraction period were used as date criterion was satisfied. For patients who met criteria based on multiple prescriptions for opioids, the earlier of the two prescription dates was used as the date of onset of “moderate-to-severe OA” |
| **Inadequate response to pain-related medications** | - Medical note of inadequate pain relief, intolerable to medication(s) or persistent pain; ***OR*** - Total or partial knee/hip joint replacement; ***OR*** - <90 days between episodes of HA or IA steroid treatment*; ***OR*** - 2 or more consecutive pain scores ≥4 or verbal rating of moderate-to-severe pain (i.e., persistency of moderate-to-severe pain) (date of the second pain assessment was used to establish persistency/worsening). - A pain scores ≥4 or verbal rating of moderate-to-severe pain (i.e., persistency of moderate-to-severe pain) following an HA or IA injection) (date of the pain assessment was used to establish persistency/worsening). | - First date of administration of HA/IA steroids within the second episode was used as date criterion was satisfied - Earliest date with confirmatory evidence during extraction period was used as date criterion was satisfied. For patients who meet criteria based on multiple prescriptions for opioids, the earlier of the two prescription dates was used as the date of onset of “moderate-to-severe OA” |
| Note: *HA injections can be a one-time procedure, but more often are given as a series of weekly injections across three, four, or five, weeks; HA injections following a such pattern are considered as a single treatment episode. If an HA injection is repeated more than 90 days apart from the date of last HA injection, it was considered as a new treatment episode.  IA steroids injections are usually given up to four times a year. Each IA steroids injection was treated as a treatment episode.  Abbreviations: HA = hyaluronic acid; IA = intraarticular; OA= osteoarthritis. | | |

**Supplemental Table 3. Performance of machine learning algorithms to identify patients with moderate-to-severe OA of the hip and/or knee with inadequate response to two or more pain-related medications applied to EMR data**

| **Metric** | **Logistic Regression** | **Classification and Regression Tree** | **Random Forest** |
| --- | --- | --- | --- |
| Positive Predictive Value (SD)† | 0.87 (0.02) | 0.88 (0.05) | 0.89 (0.02) |
| Negative Predictive Value | 0.38 | 0.46 | 0.62 |
| Sensitivity | 0.68 | 0.78 | 0.88 |
| Specificity | 0.65 | 0.65 | 0.64 |
| Accuracy | 0.68 | 0.75 | 0.83 |
| Area Under the Curve | 0.67 | 0.72 | 0.76 |
| F1 score‡ | 0.77 | 0.83 | 0.89 |

Note: *OA population of interest: patients who meet all three relevant criteria (i.e., OA of the hip and/or knee, moderate-to-severe OA, inadequate response to ≥ 2 pain-related medications).

†The means and SD are estimated from the outer folds of the nested cross validation.

‡The F1 score is calculated as the weighted average of PPV and sensitivity. A value between 0 and 1 with 1 being the highest (most accurate).

Abbreviations: EMR = electronic medical records; SD = standard deviation

**Supplemental Figure 1. Shapley Additive exPlanations (SHAP) diagram of feature importance from random forest to identify patients with moderate-to-severe OA of the hip and/or knee with inadequate response to two or more pain-related medications using claims data**


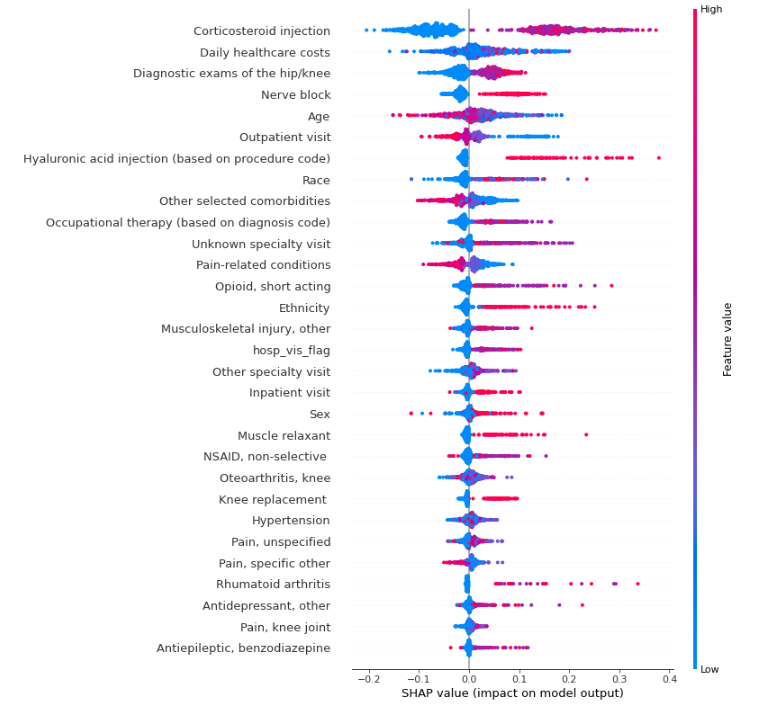


Note: The summary plot combines feature importance with feature effects. Feature names are ordered according to their importance. On the x-axis is the SHAP value which indicates how much is the impact on model output. The color represents the value of the feature from low (blue color) to high (red color). Each point on the summary plot is a SHAP value for a feature and one patient. Overlapping points are jittered in the y-axis direction, to illustrate the distribution of the SHAP values per feature. For example, a higher number of orthopedic visits (red/purple points) leads to a higher chance of being classified as case (i.e., patients with all three osteoarthritis characteristics). A lower number of orthopedic visits (blue points) leads to a lower chance of being classified as case.

**Supplemental Figure 2. Feature of importance ranking based on mean absolute Shapley Additive exPlanations (SHAP) values from random forest model to identify patients with moderate-to-severe OA of the hip and/or knee with inadequate response to two or more pain-related medications using EMR data**


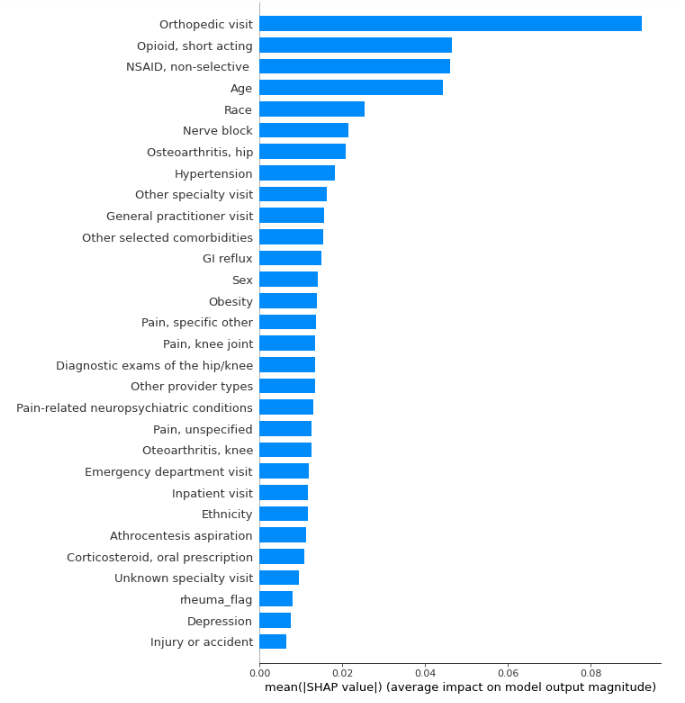


Abbreviations: EMR = electronic medical records; GI = gastrointestinal.

**Supplementary Figure 3. Shapley Additive exPlanations (SHAP) diagram of feature importance from random forest to identify patients with moderate-to-severe OA of the hip and/or knee with inadequate response to two or more pain-related medications using EMR data**


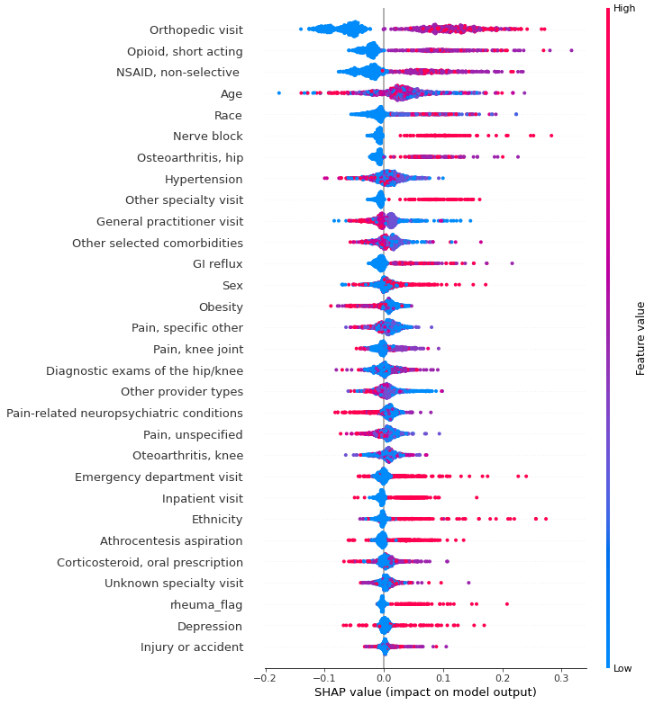


Note: The summary plot combines feature importance with feature effects. Feature names are ordered according to their importance. On the x-axis is the SHAP value which indicates how much is the impact on model output. The color represents the value of the feature from low (blue color) to high (red color). Each point on the summary plot is a SHAP value for a feature and one patient. Overlapping points are jittered in the y-axis direction, to illustrate the distribution of the SHAP values per feature. For example, a higher number of orthopedic visits (red/purple points) leads to a higher chance of being classified as case (i.e., patients with all three osteoarthritis characteristics). A lower number of orthopedic visits (blue points) leads to a lower chance of being classified as case.

Abbreviation: EMR = electronic medical records
