# Supplementary material for: Use of electronic health data to identify patients with moderate-to-severe osteoarthritis of the hip and/or knee and inadequate response to pain medications
Source: BMC Med Res Methodol. 2023 Jun 30;23:156. doi: 10.1186/s12874-023-01964-y (PMC10311749; doi:10.1186/s12874-023-01964-y)
Supplement: Supplementary file 2 — Additional file 2. Supplementary Information on Machine Learning Methods. [file 12874_2023_1964_MOESM2_ESM.docx]

## Additional File 2. Supplementary Information on Machine Learning Methods

### Nested Cross-validation

The nested cross-validation approach was used as both claims and EMR datasets presented a relatively low sample size. This procedure helped reserve data for independent tests and was necessary because hyperparameter tuning needed to be independent of model evaluation. The nested CV process ensured that no information leakage was possible for model evaluation in each outer fold, making the training method virtually completely unbiased. Additionally, the nested cross-validation approach allowed using each patient for training and validating the hyperparameters. We used the commonly accepted value of k=10 for the outer loop and k=5 for the inner loop. We compared each patient’s predicted OA status (i.e., case or comparison) based on each ML method to their true status and assessed the performance of each algorithm using sensitivity, specificity, positive predictive value (PPV), negative predictive value (NPV), and accuracy presented in the table below.

**Statistics for Evaluating Algorithm Performance**

| **Statistic** | **Definition** | **Equation** |
| --- | --- | --- |
| Sensitivity | The ratio of the total number of patients who are TP to the total number of patients who are either TP or FN | $\frac{TP}{TP+FN}$ |
| Specificity | The ratio of the total number of patients who are TN to the total number of patients who are either TN or FP | $\frac{TN}{TN+FP}$ |
| Negative Predictive Value | The ratio of the total number of patients who were TN to the total number of patients who were either TN or FN | $\frac{TN}{TN+FN}$ |
| Positive Predictive Value | The ratio of the total number of patients who were TP to the total number of patients who were either TP or FP | $\frac{TP}{TP+FP}$ |
| Accuracy | The total number of patients who are either TP or TN to the total number of patients in the cohort | $\frac{TP+TN}{TP+FP+FN+TN}$ |
| F1 | The weighted average of PPV and sensitivity. A value between 0 and 1 with 1 being the highest (most accurate). | $\frac{(2*Sensitivity*PPV)}{(Sensitivity+PPV)}$ |
| Area Under the Curve | The area under the receiver operating characteristic (ROC) curve, which plots the sensitivity against 1-specificity at various threshold settings(17) | $\int_{0}^{1} f(x)dx$, where (x, f(x)) refers to any point on the ROC curve |

Abbreviations: FN = number of false negatives; FP = number of false positives; ROC = receiver operating characteristic; TN = number of true negatives; TP = number of true positives.

### Parameter Tuning

To find the best configuration of parameters, different parameters were tested for logistics regression, classification and regression tree (CART) and random forest (RF) including:

- Maximum tree depth∈ [none, 10, 12, 15]
- Number of trees ∈ [100, 500, 1000]
- Split quality criterion ∈ [Gini impurity, information gain]
- Logistic regression: Classifier solver ['newton-cg', 'lbfgs', 'liblinear', 'sag', 'saga']
- CART: Classifier criterion ['gini', 'entropy'], Classifier maximum features [None, 'auto', 'sqrt', 'log2'], Maximum depth [none, 10, 12, 15]
- RF: Classifier criterion ['gini', 'entropy'], Classifier maximum features: ['auto', 'sqrt', 'log2'], Classifier estimators: [100, 500, 1000], Maximum depth [none, 10, 12, 15]

### Data Structure Documentation

Claims and electronic medical records (EMR) data were organized on the patient-record level, with each record representing one recorded medical encounter per patient. Flag variables for patient demographic (e.g., age, race, ethnicity) and clinical (i.e., obesity, depression, anxiety, and other comorbidities) characteristics were derived, as well pain- and osteoarthritis-related treatments (e.g., surgical procedures, corticosteroids injections, prescriptions of pain medications) and the use of healthcare resources (e.g., outpatient visit, emergency department visit) and morbidity aids. Flags for chronic conditions (e.g., anxiety, epilepsy, liver disease, diabetes) are coded as 0 (“No”) before the chronic condition occurs, and 1 (“Yes”) for all records thereafter; flags for visits, medications and surgical procedures are coded as 1 on the days they occur and 0 on all other records. In claims data, a variable representing the total daily cost of healthcare resource use was derived (*day_cost*); this variable is not available in the EMR databases. In addition to the variables described above, the datasets contain the patient ID (*studyid*), the date of the record (*service_date*), and an integer variable representing the number of months (rounded up) since the start of the patient’s data (*month*).

Classification results from chart review adjudication served as the benchmark (i.e., the true cases and comparators). This dataset contained the patient ID (*studyid*) and a flag variable representing the results (*case_flag*). Patients who were determined by the chart review to be a case are coded as 1 and patients who were determined to be a comparator are coded as 0.
