# Supplementary material for: Use of electronic health data to identify patients with moderate-to-severe osteoarthritis of the hip and/or knee and inadequate response to pain medications
Source: BMC Med Res Methodol. 2023 Jun 30;23:156. doi: 10.1186/s12874-023-01964-y (PMC10311749; doi:10.1186/s12874-023-01964-y)
Supplement: Supplementary file 4 — Additional file 4. Final Predictive Features (Pickle File), Claims Data. [file 12874_2023_1964_MOESM4_ESM.docx]

## Additional File 4. Final Predictive Features (Pickle File), Claims Data
